# Retraction: A face image classification method of autistic children based on the two-phase transfer learning

**DOI:** 10.3389/fpsyg.2026.1862576

**Published:** 2026-05-04

**Authors:** Frontiers Editorial Office

The journal retracts on the 31st August 2023 article cited above.

Following publication, concerns were raised regarding the dataset used in this article. The dataset is described as containing images of children with autism spectrum disorder (ASD), however, the images appear to have been gathered from the internet without documented clinical histories or confirmation of an ASD diagnosis. There is also no documented evidence of ethical oversight or consent from the children or from their parents or legal guardians. As a result of these concerns, and in line with Frontiers' policies and those of COPE, the publisher no longer has confidence in the results and conclusions of this article.

This retraction was approved by the Chief Editors of Frontiers in Psychology and the Chief Executive Editor of Frontiers. The authors do not agree with this retraction.

